# The conserved Lysine69 residue plays a catalytic role in *Mycobacterium tuberculosis *shikimate dehydrogenase

**DOI:** 10.1186/1756-0500-2-227

**Published:** 2009-11-16

**Authors:** Valnês S Rodrigues, Ardala Breda, Diógenes S Santos, Luiz A Basso

**Affiliations:** 1 Centro de Pesquisas em Biologia Molecular e Funcional (CPBMF), Instituto Nacional de Ciência e Tecnologia em Tuberculose (INCT-TB), Pontifícia Universidade Católica do Rio Grande do Sul (PUCRS); Porto Alegre, Brazil; 2 Programa de Pós-Graduação em Biologia Celular e Molecular, Pontifícia Universidade Católica do Rio Grande do Sul (PUCRS); Porto Alegre - RS, Brazil

## Abstract

**Background:**

The shikimate pathway is an attractive target for the development of antitubercular agents because it is essential in *Mycobacterium tuberculosis*, the causative agent of tuberculosis, but absent in humans. *M. tuberculosis aroE*-encoded shikimate dehydrogenase catalyzes the forth reaction in the shikimate pathway. Structural and functional studies indicate that Lysine69 may be involved in catalysis and/or substrate binding in *M. tuberculosis *shikimate dehydrogenase. Investigation of the kinetic properties of mutant enzymes can bring important insights about the role of amino acid residues for *M. tuberculosis *shikimate dehydrogenase.

**Findings:**

We have performed site-directed mutagenesis, steady-state kinetics, equilibrium binding measurements and molecular modeling for both the wild-type *M. tuberculosis *shikimate dehydrogenase and the K69A mutant enzymes. The apparent steady-state kinetic parameters for the *M. tuberculosis *shikimate dehydrogenase were determined; the catalytic constant value for the wild-type enzyme (50 s^-1^) is 68-fold larger than that for the mutant K69A (0.73 s^-1^). There was a modest increase in the Michaelis-Menten constant for DHS (K69A = 76 μM; wild-type = 29 μM) and NADPH (K69A = 30 μM; wild-type = 11 μM). The equilibrium dissociation constants for wild-type and K69A mutant enzymes are 32 (± 4) μM and 134 (± 21), respectively.

**Conclusion:**

Our results show that the residue Lysine69 plays a catalytic role and is not involved in substrate binding for the *M. tuberculosis *shikimate dehydrogenase. These efforts on *M. tuberculosis *shikimate dehydrogenase catalytic mechanism determination should help the rational design of specific inhibitors, aiming at the development of antitubercular drugs.

## Background

Tuberculosis (TB) remains a major global health concern. It has been estimated that one-third of the world population is infected with *Mycobacterium tuberculosis*, the causative agent of TB, and that 30 million people died from this disease in the last decade [1]. The epidemic of the human immunodeficiency virus, the increase in the homeless population, and the decline in health care structures and national surveillance are contributing factors to TB resurgence. Inappropriate treatment regimens and patient noncompliance in completing the therapies are associated with the emergence of multi-drug resistant TB (MDR-TB), defined as strains of *M. tuberculosis *resistant to at least isoniazid and rifampicin, two pivotal drugs used in the standard treatment of TB [2]. It has been reported the emergence of extensively drug-resistant (XDR) TB cases, defined as cases in persons with TB whose isolates are MDR as well as resistant to any one of the fluoroquinolone drugs and to at least one of the three injectable second-line drugs [3]. XDR-TB is widespread raising the prospect of virtually incurable TB worldwide [3]. There is thus an urgent need for new drugs to improve the treatment of MDR- and XDR-TB, and to provide more effective drugs to shorten the duration of TB treatment. Enzyme inhibitors make up roughly 25% of the drugs marketed [4], and are thus important promising drug targets.

**Figure 1:**
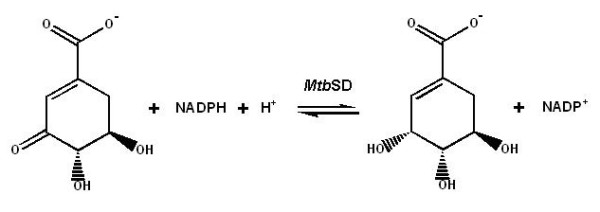
**The shikimate dehydrogenase-catalyzed reaction**.

The shikimate pathway is an attractive target for the development of herbicides and antimicrobial agents because it is essential in algae, higher plants, bacteria, and fungi, but absent from mammals [5]. The mycobacterial shikimate pathway leads to the biosynthesis of precursors of aromatic amino acids, naphthoquinones, menaquinones, and mycobactins [6], and is essential for *M. tuberculosis *viability [7]. Shikimate dehydrogenase (SD; EC 1.1.1.25), the fourth enzyme of this pathway, catalyzes the NADPH-dependent reduction of 3-dehydroshikimate (DHS) to shikimate (SHK, Fig. 1). We have previously reported production, characterization, and determination of kinetic and chemical mechanisms of *aroE*-encoded SD from *M. tuberculosis *H37Rv strain (*Mtb*SD) [8-10]. Multiple sequence alignment, comparative homology modeling, and pH-rate profiles suggested that the Lysine69 in the DHS/SHK binding site of *Mtb*SD plays a role in substrate binding and/or catalysis [10,11]. Here we describe site-directed mutagenesis, steady-state kinetics, fluorimetric measurements and structural analyses to probe the role of Lys69 in *Mtb*SD and provide insight into the molecular basis of DHS/SHK recognition and/or catalysis.

## Methods

### Site-directed mutagenesis

A previously constructed pET-23a(+)::*aroE *recombinant vector [8] was used as a template for PCR-based mutagenesis using the Quick Change site-directed mutagenesis kit (Stratagene, La Jolla, CA). The synthetic oligonucleotides employed were as follows: 5'-ggtgtttcggtgaccatgccgggc**gcg**ttcgccgccctgcggttcg-3' (forward) and 5'-cgaaccgcagggcggcgaa**cgc**gcccggcatggtcaccgaaacacc-3' (reverse) (in bold is the codon for alanine). *Pfu*Turbo^® ^DNA polymerase and standard PCR amplification program were employed. The PCR product was treated with *Dpn*I endonuclease that specifically digests the methylated DNA template, and selects for the mutation-containing synthesized DNA.

### Expression, release and purification of recombinant MtbSD

The recombinant plasmid was introduced into *E. coli *C41 (DE3) host cells (Novagen, Madison, WI) by electroporation. Single colonies were used to inoculate 2 L of LB medium containing 50 μg mL^-1 ^ampicillin, and 1 mM isopropyl β-D-thiogalactopyranoside was added to cultures reaching an OD_600 _of 0.4 - 0.6, and grown for 24 h at 37°C at 180 rpm. Cells (5 g) were harvested by centrifugation at 14,900 *g *for 30 min at 4°C, and stored at -20°C. The freeze-thaw method was used to release the proteins in the soluble fraction [8]. The purification protocol was as previously described [12]. Samples of the purification steps were analyzed by SDS-PAGE [13] and protein content by the Bradford's method [14].

### Enzyme activity assays and determination of kinetic parameters

**Figure 2:**
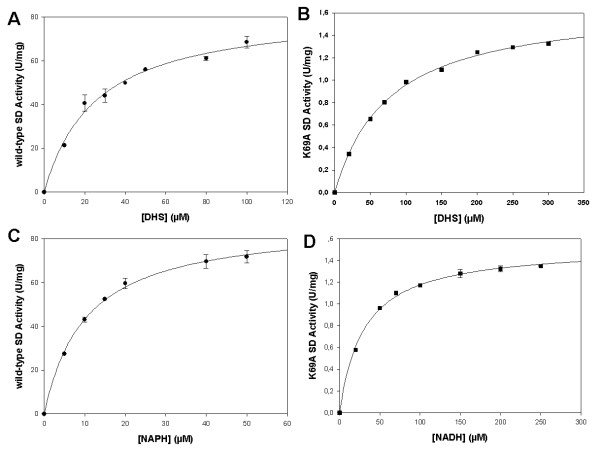
**Steady-state kinetic measurements for wild-type (A and C) and K69A (B and D) *Mtb*SD in the forward direction**. A and B: DHS concentrations were varied while NADPH concentration was maintained at a fixed saturating level. C and D: NADPH concentrations were varied while DHS concentration was maintained at a fixed saturating level.

Steady-state kinetics measurements of homogeneous *Mtb*SD K69A mutant activity were carried out for the forward direction at 25°C in 100 mM potassium phosphate buffer, pH 7.3, by monitoring the decrease in absorbance at 340 nm (ε = 6220 M^-1 ^cm^-1 ^for NADPH) accompanying the conversion of NADPH and DHS to NADP^+ ^and SHK. The K69A activity was measured at varying final concentrations of DHS (20-300 μM) and NADPH at constant saturating level (200 μM); and varying NADPH concentrations (20-250 μM) and DHS at constant saturating level (250 μM; Fig. 2B and 2D). The wild-type SD activity was measured at various DHS concentrations (10-100 μM) and NADPH at constant saturating level (200 μM); and various NADPH concentrations (5-50 μM) and DHS at constant saturating level (250 μM; Fig. 2A and 2C). All measurements were in duplicate. Steady-state kinetic constants were obtained by non-linear regression analysis of the kinetic data fitted to the the Michaelis-Menten equation (v = V_max _× [S]/K_m _+ [S]) using the SigmaPlot software (SPSS, Inc).

### Fluorescence spectroscopy

Fluorescence titration was performed in a Shimadzu spectrofluorophotometer RF-5301PC at 25°C by making microliter additions of DHS substrate stock solutions to 2 mL of 1 μM of *Mtb*SD in 100 mM potassium phosphate buffer pH 7.3. The binding of DHS to *Mtb*SD causes a quench in the intrinsic protein fluorescence (λ_exc _= 300 nm; 310 ≤ λ_em _≤ 450 nm; maximum λ_em _at 340 nm), which allowed monitoring of *Mtb*SD-DHS binary complex formation at equilibrium. To assess the DHS inner-filter effect in the fluorimeter, two cuvettes were placed in series so that the contents of the first cuvette acted as a filter of the excitation light and the light emitted from the second cuvette detected. DHS was added to the first cuvette, while the second cuvette contained the protein. In this manner, DHS inner-filter effect on protein fluorescence could be assessed. The results were plotted to a rectangular hyperbola by using the nonlinear regression function of SigmaPlot 2004 (SPSS, Inc.).

### MtbSD K69A structural analysis

The wild-type *Mtb*SD three-dimensional (3D) model was built previously by comparative homology modeling using *Escherichia coli *SD as template (**PDB ID**: 1NYT) [10], however, a more recent 3D structure for *Thermus thermophilus *SD (**PDB ID**: 2EV9), solved by X-ray crystallography at 1.90 Å in complex with shikimate [15], was used as a template for *Mtb*SD K69A mutant modeling, as well as to evaluate DHS/SHK substrate binding mode to *Mtb*SD. *T. thermophilus *SD shows higher primary structure identity to *Mtb*SD (~30% identity and 13% strong similarity) than *E. coli *SD (26% identity and 15% strong similarity), and the presence of both enzyme's cofactor and substrate bound at its active site makes such structure a more suitable template for *Mtb*SD comparative homology modeling.

Target and template pair-wise sequence alignment required small gaps inclusion on both *M. tuberculosis *and *T. thermophilus *SD primary sequences. *Mtb*SD K69A model was built by restrained-based homology modeling implemented in MODELLER9v1 [16], with the standard protocol of the comparative protein structure modeling methodology, by satisfaction of spatial restrains [17,18]. Atomic coordinates of SHK heteroatoms were copied from template structure into the *Mtb*SD K69A model. The best model was selected according to MODELLER objective function [19] and subjected to energy minimization for amino acid side chain and main chain rearrangements with GROMACS package [20] using the 43a1 force-field. The system was submitted to an initial steepest descent energy minimization *in vacuo *with a maximum number of 400 minimization steps, followed by a maximum of 3000 steps of conjugate gradient energy minimization.

The program PROCHECK and VERIFY 3D were employed to, respectively, analyze stereochemical quality and validate the 3D profile of the model, as previously described [10]. Structural correspondence between *Mtb*SD K69A model and *T. thermophilus *was evaluated by their root-mean square deviation (RMSD). H-bond interactions were evaluated with LIGPLOT v4.4.2 [21], considering an atomic distance cut off of 3.9 Å (program default values).

## Findings and Discussion

### Site-directed mutagenesis, protein expression and purification

Total sequencing of *aroE *mutant DNA into pET-23a(+) vector confirmed that the mutation was introduced into the expected site and that no unwanted mutations were introduced by the PCR amplification step. The recombinant *Mtb*SD protein purification protocol resulted in a protein yield of 13%, according to the previous protocol [12].

### Steady-state kinetic parameters

The apparent steady-state kinetic parameters for K69A (Fig. 2B and 2D) and wild-type *Mtb*SD (Fig. 2A and 2C) are given in Table 1. It is noteworthy that the catalytic constant (k_cat_) value for wild-type *Mtb*SD (50 s^-1^) is 68-fold larger than K69A (0.73 s^-1^), whereas there was a modest increase in the K_m _values for DHS (K69A = 76 μM; wild-type *Mtb*SD = 29 μM) and NADPH (K69A = 30 μM; wild-type *Mtb*SD = 11 μM). The apparent second-order rate constant (k_cat_/K_m_) values for DHS (K69A = 9.6 × 10^3 ^M^-1^s^-1^; wild-type *Mtb*SD = 1.7 × 10^6 ^M^-1^s^-1^) and NADPH (K69A = 24 × 10^3 ^M^-1^s^-1^; wild-type *Mtb*SD = 4.5 × 10^6 ^M^-1^s^-1^) indicate that the mutant has a lower specificity constant for both substrates. A comparison of k_cat_/K_m _values is an appropriate method to assess the effect(s) of a mutation on substrate(s) binding and catalysis since it includes the activation energies and the binding energies [22]. A change of 12.8 kJ mol^-1 ^can be calculated when we compare the values of k_cat_/K_m _of DHS reduction for wild-type and K69A *Mtb*SD. These results indicate that the conserved Lys69 residue in *Mtb*SD plays a critical role in catalysis, but plays no role in substrate binding. Moreover, the fact that the K69A *Mtb*SD mutant protein still binds DHS and NADPH with only slightly larger K_m _values as compared to wild-type enzyme indicates that the mutant protein is properly folded giving more confidence that the results are not an artifact generated by the mutation. Linearity of each measurement and dose dependence when adding different volumes of the enzyme solution were confirmed for all enzyme activity assays (data not shown).

**Table 1:** Apparent steady-state kinetic parameters and equilibrium binding constants for wild type and K69A mutant *Mtb*SD

Parameter	Wild-type	K69A
V_max _(U mg^-1^)^a^	110 ± 2	1.61 ± 0.03
K_m _DHS (μM)^a^	29 ± 2	76 ± 4
K_m _NADPH (μM)^a^	11.0 ± 0.6	30 ± 2
k_cat _(s^-1^)^a^	50 ± 1	0.73 ± 0.01
k_cat_/K_m _DHS (M^-1 ^s^-1^)^a^	1.7 (± 0.1) × 10^6^	9.6 (± 0.5) × 10^3^
k_cat_/K_m _NADPH (M^-1 ^s^-1^)^a^	4.5 (± 0.2) × 10^6^	24 (± 2) × 10^3^
K_d _DHS (μM)^b^	32 ± 4	134 ± 21

^a ^steady-state kinetic parameters

^b ^spectroscopic measurements of intrinsic protein fluorescence (equilibrium binding)

### Equilibrium binding

**Figure 3:**
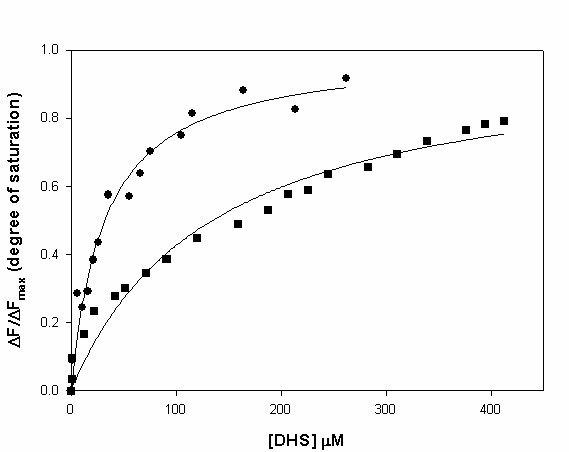
**Spectroscopic measurements of intrinsic protein fluorescence for wild-type (black circles) and K69A (black squares) *Mtb*SD upon DHS binding at varying concentrations**.

Spectrofluorimetric assays were carried out to determine the equilibrium dissociation constant for *Mtb*SD-DHS binary complex formation (Fig. 3). The change in the intrinsic protein fluorescence at varying DHS concentrations (2-450 μM) yielded equilibrium dissociation constant (K_d_) values of 32 (± 4) μM for wild-type *Mtb*SD and 134 (± 21) μM for K69A mutant (Table 1). Interestingly, measurements of changes in nucleotide fluorescence upon NADPH binding to wild-type *Mtb*SD (λ_exc _= 370 nm; 380 ≤ λ_em _≤ 600 nm; maximum λ_em _at 445 nm) did not show any saturation, which indicates a very large K_d _value. This is consistent with a steady-state ordered bi-bi mechanism with DHS binding first followed by NADPH binding to *Mtb*SD active site [10]. Moreover, these experiments confirmed our proposal that the Lys69 residue plays a minor role, if any, in substrate binding.

### Structural analysis

Two crystallographic structures of SD in complex with both its coenzyme and SHK (*Aquifex aeolicus*, **PDB ID**: 2HK9[23] and *T. thermophilus*, **PDB ID**: 2EV9[15], solved by X-ray diffraction at 2.20 Å and 1.90 Å resolution, respectively) are available in the Protein Data Bank (PDB). *T. thermophilus *SD structure was chosen as template for comparative homology modeling of *Mtb*SD K69A and for SHK binding analysis because of larger primary sequence identity to *Mtb*SD (~30% identity and 13% strong similarity) as compared to *A. aeolicus *(24% identity and 16% strong similarity), and lower requirement for inclusion of gaps in primary sequence comparisons.

Analysis of the 3D structures of SDs in complex with NADP^+ ^and SHK suggest that Lys64 in *T. termophilus *[15] and Lys70 in *A. aeolicus *[23] interact with C3 of DHS and act as an acid-base catalytic group. Corresponding Lys residue is also suggested to be catalytically active in *Staphylococcus epidermidis *[24], *Haemophilus influenzae *[25] and *Arabidopsis thaliana *[26] SDs.

**Figure 4:**
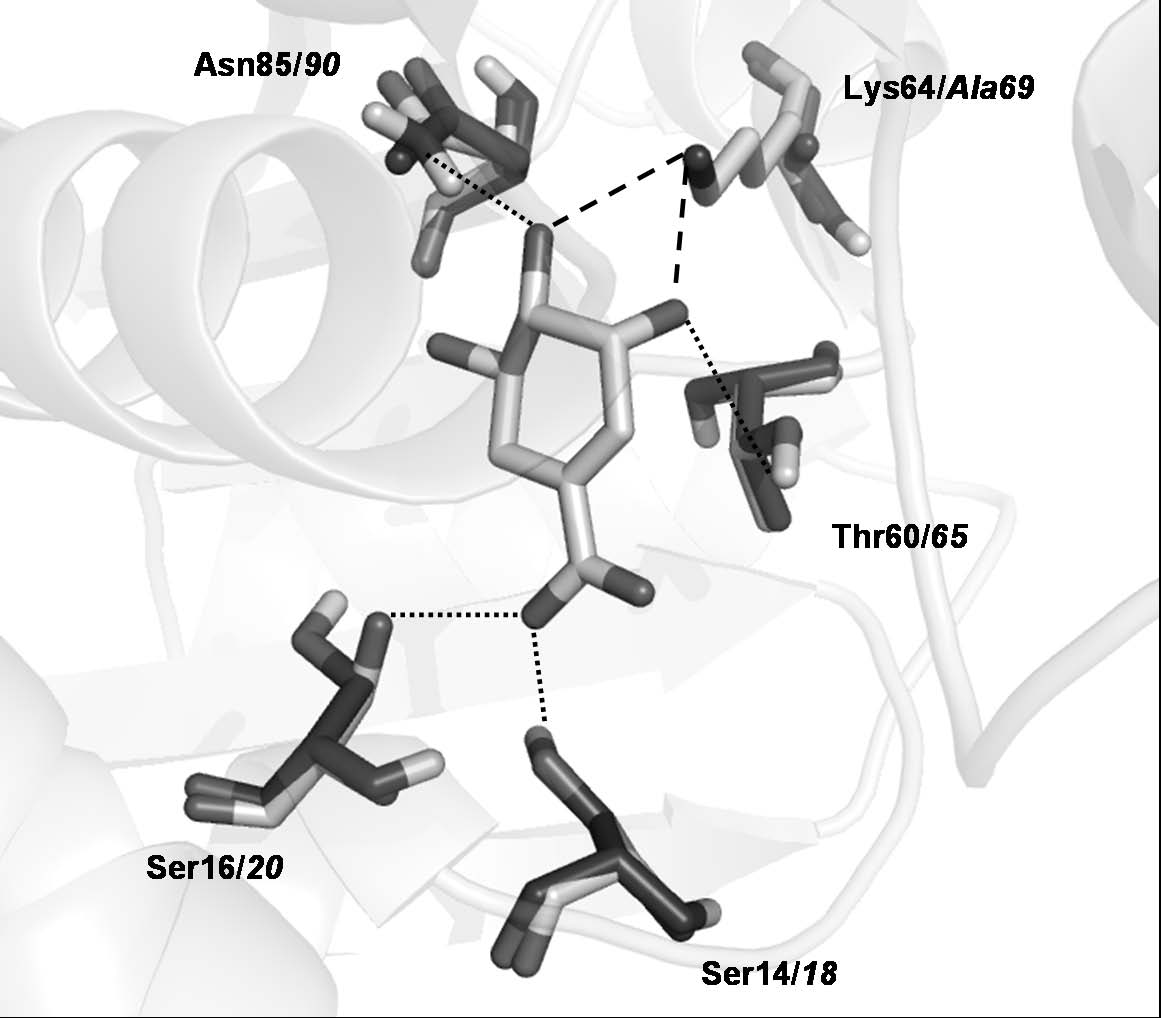
***Mtb*SD K69A model superimposed on experimentally solved *T. thermophilus *SD structure**. Amino acid side chains involved in SHK binding and SHK molecule are shown as sticks. *T. thermophilus *and *Mtb*SD K69A amino acids are colored, respectively, in light gray (residue number in **bold**) and dark gray (residue number in *italics*). H-bonds are shown as dotted lines; dashed lines represent H-bonds between Lys**64**/*69*, missing in *Mtb*SD K69A model.

No significant changes were observed on protein's overall tertiary structure and in DHS/SHK binding site after energy minimization of the *Mtb*SD K69A model. The RMSD deviation from template of only 0.55 Å, 99% of amino acids within allowed regions of the Ramachandran's plots and PROCHECK parameters indicate that the K69A model satisfies all stereochemical requirements. Considering only substrate binding site amino acids, *Mtb*SD K69A RMSD values reduced to 0.12 Å when compared to template. Such minor structural variations indicate a strong conservation of the amino acid arrangement at the enzyme's substrate binding site, not only at their α-carbon position but also in regard to their χ rotational angles, where only *Mtb*SD residue Ser20 (Ser16 on template) shows variation on its rotational angle χ_1 _(2.02 Å for O**γ **atom), although not affecting H-bonding to SHK. (Fig. 4).

### H-bonding pattern between MtbSD and SHK

LIGPLOT analysis showed that the pattern of H-bonding to SHK is conserved in *T. thermophilus *SD and *Mtb*SD K69A (Table 2). Gln243 residue in *M. tuberculosis *(corresponding to Gln235 in *T. thermophilus *SD) also makes a hydrophobic contact to C5 of SHK, as observed on template's structure. No significant amino acid rearrangements were observed for *Mtb*SD K69A. Not surprisingly, the noticeable change is the loss of two H-bonds with the SHK molecule in K69A mutant. It could be argued that the loss of two H-bonds in K69A *Mtb*SD mutant would demonstrate the role of Lys69 in DHS/SHK binding. The energies of hydrogen bonds have been variously estimated to be between 12 and 38 kJ mol^-1 ^[22]. Spectrofluorimetric measurements showed a reduction of 3.5 kJ mol^-1 ^in binding energy of DHS to K69A *Mtb*SD as compared to the wild-type enzyme.

**Table 2:** Hydrogen bonding pattern of SD enzyme and SHK substrate. *T. thermophilus *values are shown in bold, *Mtb*SD K69A corresponding values are shown in *italics*.

SD residue	SD Atom	SHK atom	H bond distance (Å)
**Ser14**/*Ser18*	**Oγ**	**O2**	**2.62**/*2.55*
**Ser16**/*Ser20*	**Oγ**	**O2**	**2.72**/*2.73*
**Thr60**/*Thr65*	**Oγ 1**	**O11**	**3.28**/*3.22*
**Lys64**/*Lys69*	**Nζ**	**O11**	**2.80**/--
**Lys64**/*Lys69*	**Nζ**	**O12**	**3.10**/--
**Asn85**/*Asn90*	**Nδ 2**	**O12**	**3.15**/*2.93*

Atom numbering as [24]*.

* O2 corresponds to carboxyl negative oxygen atom, O11 to hydroxyl oxygen bound to C3, O12 to hydroxyl oxygen bound to C4, and O7 to hydroxyl oxygen bound to C5, as depicted in Fig. 1.

## Conclusion

Based on double isotope effects and pH-rate profiles, we have previously proposed that the chemical mechanism for *Mtb*SD involves hydride transfer and solvent proton transfer in a concerted manner, and that an amino acid residue with an apparent pK_a _value of 8.9 is involved in catalysis [10]. Here we demonstrate that the *Mtb*SD Lys69 is important for catalysis and is likely involved in stabilization of the developing negative charge at the hydride-accepting C-3 carbonyl oxygen of DHS for the forward reaction, acting as an acid-base catalytic group that donates a proton to DHS carbonyl group during reduction, playing a minor role, if any, in substrate binding.

In bacteria, four subclasses of SD have been identified, distinguished by their phylogeny and biochemical activity [27]. In agreement with our results, mutagenesis studies showed that the corresponding residues Lys67 in *Haemophilus influenzae *SD [25] and Lys385 in *Arabidopsis thaliana *dehydroquinate dehydratase-SD [26] play a critical role in catalysis and no role in substrate binding. Interestingly, site-directed mutagenesis of *Escherichia coli *YdiB (a bifunctional enzyme that catalyzes the reversible reductions of 3-dehydroquinate to quinate and 3-dehydroshikimate to shikimate using as co-substrate either NADH or NADPH) has shown that the conserved Lys71 residue plays a primary role in substrate binding in the Michaelis complex and, although it contributes to some extent to transition state stabilization, plays no essential role in catalysis [28].

It should be pointed out that mechanistic analysis should always be a top priority for new enzyme-targeted drug programs since effective enzyme inhibitors take advantage of enzyme chemistry to achieve inhibition [29]. Accordingly, we hope that the results here presented will pave the way for the target-based rational design of novel effective antitubercular agents. The design of inhibitors of *Mtb*SD enzyme activity may contemplate derivatization of C-3 of DHS with functional groups that make strong interactions with the Lys69 side chain. It would appear to be unlikely that a mutation of Lys69 residue would be selected for to afford drug resistance because here is shown that this amino acid residue plays a critical role in *Mtb*SD enzyme activity. However, an important additional point to be considered when selecting a particular enzyme as a successful drug target is to determine a flux control coefficient, which measures the sensitivity of flux to a change in enzyme concentration. An enzyme is likely a good target if the flux control coefficient is high, whereas it is less likely a good target if the flux control coefficient is close to zero (virtually all the activity must be eliminated by an inhibitor to have the desired chemotherapeutic effect). Bifunctional dehydroquinate dehydratase-shikimate dehydrogenases in plants are responsible for preferential routing of carbon to aromatic amino acid synthesis [30]. However, how shikimate dehydrogenase affects the shikimate pathway flux in *M. tuberculosi*s is still unknown. Therefore, strategies for drug design contemplating derivatization of C-3 of DHS should also take into account whether shikimate dehydrogenase has a low flux control coefficient because mutation of Lys69 would be one way of gaining resistance to this enzyme's inhibitors.

## List of abbreviations used

TB: tuberculosis; MDR: multi-drug resistant; XDR: extensively drug-resistant; *Mtb*SD: shikimate dehydrogenase from *M. tuberculosis*; DHS: 3-dehydroshikimate; SHK: shikimate; K_m_: Michaelis-Menten constant; RMSD: root-mean square deviation; k_cat_: catalytic constant; K_d_: equilibrium dissociation constant.

## Competing interests

The authors declare that they have no competing interests.

## Authors' contributions

VSRJ performed most of the experiments and drafted the manuscript. AB performed the structural analysis and helped to write the manuscript. The study was conceived and coordinated by DSS and LAB, who have also helped to draft the manuscript. All authors read and approved the final version of the manuscript.
